# Biotechnology in China – regulation, investment, and delayed commercialization

**DOI:** 10.1080/21645698.2022.2068336

**Published:** 2022-05-04

**Authors:** Zhihua Xiao, William A. Kerr

**Affiliations:** a College of Economics and Management, Jiangxi Agricultural University, Nanchang, Jiangxi, China; b Department of Agricultural and Resource Economics, University of Saskatchewan, Saskatoon, Saskatchewan, Canada

**Keywords:** China, commercialization, GMOs, regulation, social cohesion

## Abstract

China has been investing heavily in biotechnology to increase agricultural productivity. While a number of Chinese developed GM crops have cleared the required scientific hurdles – some more than a decade ago – commercialization has not been approved. The regulatory regime for GMOs in China is relatively less well understood than that of the US or the EU. This paper provides a systematic overview of China’s regulatory regime, R&D investment and delayed commercialization decisions on biotechnology over the last 40 years and draws some conclusions regarding the likelihood of the commercialization for major GM crops in the future.

## 1. Introduction

When crops developed through the use of modern agricultural biotechnology were first grown commercially in the mid-1990s they were touted as having the potential to increase agricultural productivity to a significant degree and be a major pillar in the struggle to feed nine billion people by 2050.^1^ Since that optimistic time, the use of the technology has been mired in controversy, divergent regulatory trajectories, barriers to international trade and unrealized potential in scientific innovation.^2^ The United States, China and the European Union (EU), the world largest agricultural producers and the most important trade economies, have developed very different policy systems for biotechnology. Policy in the US is broadly supportive, while EU’s policy is precautionary. China’s policy is bifurcated exhibiting both supportive and precautionary facets. While much has been written about the policies pertaining to biotechnology in the US and the EU,^3^ China’s policy has been less well-understood relative to those in western countries.

After 40 years of rapid economic growth and structural transformation in China, agriculture faces critical challenges regarding increasingly contaminated arable land, water shortages, and the rising cost of farm labor and land. In 2004, China moved from being a net exporter of food to a net importer^4^ and the import-export gap has been expanding ever since. Food security risk is a deep-rooted concern of the Chinese government given the large population and the experience of periods of poor food security in the past, including major famines. As food insecurity issues can be a cause of political instability^5^ and a perception that food can be used as political weapon by foreign countries to threaten a nation’s security,^6^ the Chinese government, hence, perceives that increasing agricultural productivity is an essential contributor to achieving its food security goals.^7^ A failure to increase agricultural productivity, from a global view, will mean a significant increase in imports given China’s projected economic growth. Increase imports on such a scale will drive up international food prices and reduce the ability to reach global food security goals.^8^

To improve agricultural productivity, China’s government has been investing heavily in biotechnology R&D. The public R&D investment in biotechnology increased from US$26 million in 1986 to US$99 million in 2005^9,10^ and reached US$3.8 billion over the period 2008–2020.^11^^1^^1^The exchange rate of Chinese Yuan to US dollar in 2008 was applied for the calculation.

The strategic objectives for biotechnology policies are: (1) to guarantee food security and food self-sufficiency through improvements to agricultural productivity; (2) become the global leader in biotechnology by developing a competitive Chinese-owned agricultural biotechnology industry.^12–15^ Around these central objectives, a sophisticated regulatory system has been established over the last 40 years.

This paper seeks to shed new light on China’s biotechnology policy and to contrast it with those of the US and the EU. Understanding China’s regulatory regime, given the importance of its agricultural output in global production, provides insights into the potential of achieving food security goals internationally.

## 2. Comparison of Global Regulatory Regimes for Biotechnology – the EU, China, and the US

The use of genetically modified organisms (GMOs) has been a contentious issue across the globe among some members of civil societies.^16^ In the European Union (EU) four groups in civil society with strong preferences have opposed the domestic licensing of genetically modified organisms (GMOs) in agriculture and their import. The four groups were: (1) those already concern with the quality and safety of the food they were consuming (e.g. organic, pharmaceutical residues, hormones used in beef production); (2) those concerned about threats to the environment; (3) those with ethical concerns about the technology (e.g. messing with God’s work) and; (4) those concerned with agribusiness multinationals having too much power in the food system – because most biotechnology was being developed, commercialized and owned by large multinationals.^17^ The convergence of these four influential interest groups were able to prevail with policy makers in the EU culminating in a moratorium on domestic production using GMOs and a *de facto* ban on imports.^18^ Except for some relaxation of import rules for genetically modified animal feeds, the production and import bans remain in place.^19^

In the US the same debates raged in civil society, The US, however, has a long tradition of fostering productivity enhancing agricultural technologies starting with the land grant university system in the 1860s, state level research stations and research undertaken by the United States Department of Agriculture. Further, there had been considerable success associated with applied genetic research.^20,21^ Transgenic methods were treated as a continuation of that tradition and approvals followed similar procedures meaning that the first GMO-crops produced by US-based multinationals passed through the regulatory system in a timely fashion and were commercialized in the mid-1990s.^22^ While GMOs are widely consumed in the US, the debate over the technology has never entirely gone away with those opposed using a variety of tactics to try to have greater restrictions placed on their production and use.^23^ While there have been a wide range of organizations that have attempted to stifle the use of GMOs in the US, these efforts have thus far ended in failure, but those efforts continue.^2^

Other countries, including China, show a mix of regulatory forms between the polar outcomes represented by the EU and the US.^24^ After more than 25 years since the first commercialization of GM crops, many countries are still struggling to put regulatory regimes for GMOs in place, in part because it has been difficult to find a way through given the vociferousness of the debate regarding biotechnology. While governments have always had a role in the scientific development of GMOs, outside of China major investments in the development of biotechnology have been made by the private sector.^25^ In China, the government has taken the lead in investing in and developing new GMO- crops.^26^ Hence, in China, the conflict between those with reservations regarding the technology is directly with the government rather than with private sector firms investing in the development of the technology. The interplay between considerable investment by government in the development of agricultural biotechnology and concerns raised among members of civil society is not transparent but is crucial to understanding the state of play regarding commercialization of GMOs in China.

Until the end of the first decade of the 21^st^ century there appears to have been little knowledge regarding GMOs among the Chinese public and use of the technology was not an issue. Over 2002–2003, Chinese consumers’ acceptance level of GM foods was relatively higher than other countries such as the UK, Australia, and Japan.^27^ Starting in 2010, however, stories apposing biotechnology began to be manifest in the minor Chinese media. Five stories garnered considerable attention; (1) reports of sperm count declines among college students as a result of eating GM-maize produced by Monsanto^28^; (2) anomalies in animal populations – sows and ewes aborting and populations of dogs and mice declining from consuming maize incorrectly identified as being GM^29^; (3) non-GM cooking oil being used in Ministry of Agriculture (MOA) kindergartens which evolved into a general rumor that MOA cafeterias generally were eschewing GM-cooking oil – meaning MOA officials were obtaining special status^30^; (4) the Golden Rice scandal whereby on August 1, 2012, a paper in the *American Journal of Clinical Nutrition* concluded that Golden Rice, a GM rice developed by Syngenta, was a rich source of Vitamin A according to an experiment conducted among 80 Chinese children age six to eight in 2008^31^– with the latter played up by Greenpeace and others as using Chinese children as lab rats; (5) associating GMO with the formation of tumors and infertility – on June 21, 2013, the Heilongjiang Soybean Association (HSA), a non-government organization opposing GMOs, released a report suggested that GM soybeans could cause tumors and infertility.^32^ These stories, among others, garnered considerable attention and credence among consumers. They also proved difficult for authorities to refute. Eventually, the above rumors and scandals evolved into a number of influential debate themes pushed by anti-GMO activists. These were specifically designed to appeal to the morality and patriotism of Chinese society. Consequently, consumers’ acceptance level decreased and the rejection level increased dramatically^33^ with 94.5% of Chinese consumers rejecting future commercialization in 2014.^34^

The regulatory environment for GMOs in the EU, China and the US is contrasted in Table 1.

**Table 1.** The regulatory environment for GMOs in the EU, China and the US

	European Union	China	United States
Regulatory Framework	GM crops are considered as a special category	GM crops are considered as a special category	GM crops are considered the same as conventional crops
Approval process	Scientific approval first followed by political approval (but Member States can override European Commission approvals)	Scientific approval first followed by political approval prior to commercialization	Scientific approval
Mandatory Labeling	Yes	Yes	No
Imports	Not for human consumption but allowed for animal feeds	Not for human consumption but allowed for animal feeds	Yes
Investment	Private (normally foreign firms seeking approvals)	Primarily government	Private (public contribution to basic research)
Commercialization	None since 2000	No major crops since 1997	Ongoing as new varieties approved
Anti-GMO advocates	Have been effective in influencing policy makers	May have been effective in influencing policy makers	Have not been effective in influencing policy makers

## 3. The Development of the Chinese Regulatory Regime for Biotechnology

The formation of biotechnology policies in China over the last 40 years can be categorized into four stages as shown in Table 2.

**Table 2.** China’s legislation and regulations of biotechnology: 1978–2020

Year	Sector and Regulations	Main Contents pertaining to Biotechnology
**Stage I (1978–85): Build Up Regulatory Environment for Launching Biotech R&D**		
1978	MOST: *National Sci. & Tech. Development Program Outline (1978–85)*	Accumulate resources to develop 8 science fields including agriculture; Improve seed varieties for high-productivity, high quality, and pest-resistance; Develop new breeding theories and technologies; Develop theories and technologies based on genetics and agricultural germplasm.
1985	MOST, SDPC, and SETC: *National Biotechnology Development Policy Outline*	Jointly compose a draft of a biotechnology development outline, which was issued by the State Council Office in 1988.
**Stage II (1986–92): Initiate Biotech R&D**		
1986	Central Gov’t: *National High-tech Research & Development Plan* *(863 Plan)*	To improve China’s science and technology research ability in seven high-tech fields to compete with the most advanced technology in the world.
1988	SSTC: *High-tech Industrialization Development Plan (Torch Plan)*	Promote and encourage commercial use of advanced technologies funded by the 863 Plan.
**Stage III (1993–2009): Develop Bio-Administration System and Preparing to Promote Commercializing GM crops**		
1993	SSTC: *Decree 17* *Measures for the Safety Administration of Genetic Engineering*	Classify genetic engineering (GE) into 4 classes according to the extent of its risks to human beings, animals, plants, and microorganisms and the ecological environment. Regulate GE’s safety evaluation, application and approval, safety control measures, and legal responsibilities.
1996	Ministry of Agriculture (MOA): *Decree 7* *Implementation Measures for the Safety Administration of Agricultural Genetic Engineering*	According to SSTC *Decree 17*, the MOA is authorized to conduct biosafety evaluation for Ag GMOs by establishing the Administration Office of Ag GMOs (AO) and Administration Committee for Biosafety of Ag GMOs (AC).
1997	SSTC: *National Basic Research and Development Plan* *(973 Plan)*	Support a range of programs for basic R&D. Life sciences and biotechnology are the key areas to support. The total budget of 973 Program is US$302 million in 1997–2002 and more than US$238 million for life sciences and biotechnology.
	MOA: *Commercializes Bt cotton*	
2000	NPC: *Seed Law*	For the first time, biosafety administration of GM plants is included in the national law.
2001	State Council: *Decree 304*	Establish Inter-Ministerial Joint Conference for Administration of Ag GMOs Safety (IMJC) The MOA sets up Administration Office for Biosafety of Ag GMOs (AO), and Administration Committee for Biosafety of Ag GMOs (AC). Mandatory labeling for Agricultural GMOs
2002	MOA: *Decree 8 Measures of Administration on Evaluation of the Safety of Agricultural GMOs*	The safety evaluation is categorized into three groups: plants, animals, and microorganisms. The biosafety examination is a science-based stage-by-stage and case-by-case screening process. Formulate the governance structure for biosafety administration from the central government level to the county level.
	MOA: *Decree 9 Measures of Safety Administration on Import of Agricultural GMOs*	Regulate imports of Ag GMOs. The MOA and GAQSIQ make decisions on approval or disapproval of the case within 270 days since received the application.
	MOA: *Decree 10 Measures of Labeling Administration of Agricultural GMOs*	Formulate mandatory labeling of Agricultural GMOs. The MOA takes the responsibility to examine, monitor, and administrate the labeling.
	SDPC, SETC, and MOTEC: *Catalog for the Guidance of Foreign Investment Industries*	Foreign companies are forbidden to invest in breeding Agricultural GMOs and producing GM seeds.
2004	GAQSIQ: *Decree 62 Measures on Inspection and Quarantine Administration of Import & Export of GM Products*	GAQSIQ is assigned the managerial authority to examine the cross-border movement of GM plants and their products, GM animals and their products, GM microorganisms and their products, and GM foods.
	MOA: *Decree 38* Amends *Decree 8, 9, and 10* issued in 2002	Application fees are removed Reduce the response time of MOA to applicants from 3 months to 20 days The MOA conducts the safety administration for importing Agricultural GMOs Shorten the response time of labeling examination and Agricultural GMOs approval from 30 to 20 days.
2008	MOST: *Major Breeding Projects of GMOs Varieties (GMMP)*	The largest public investment specifically for GM biotechnology R&D. The aggregate public R&D budget of GM biotechnology will reach US$3.8 billion over 2008–2020.
2009	NPC: *Food Safety Law*	GM food safety is included in the national law for the first time.
	MOA: *Issues production safety certificates to GM rice and maize*	The MOA issues production safety certificates to Bt rice (Huahui-1 and Xianyou-63) and phytase maize (BVLA430101).
**Stage IV (2010 and after): Delays in Commercialization of GM Major Crops**		
2016	MOST: *NRDP*	Launch *National Key R&D Plan (NRDP)*, assemble programs of 863, 973, and others.
2019	MOA: *Issues production safety certificates to GM maize and soybean*	The MOA issues production safety certificates to IR-HT maize (DBN9936 and Ruifeng125) for the application in north spring-maize zone, and HT soybean (SHZD3201).
2020	*The ending year of GMMP*	
	MOA: *Issues production safety certificates to GM maize and soybean*	The MOA issues production safety certificates to HT maize (DBN9858) and IR-HT maize (DBN9936 and DBN9501) and HT soybean (ZH6106 and DBN9004),
2021	MOA: *Issues production safety certificates to GM maize and soybean*	The MOA issues production safety certificates to IR maize (ND207 and Ruifeng8) and IR-HT maize (DBN3601T, i.e., DBN9936× DBN9501)
2022	MOA: Amends *Measures of Administration on Evaluation of the Safety of Agricultural GMOs*	Administration of GM crops is included in regulations of normal seed varieties, for the first time in history.
	Amends *Measures of Examination for Major Crop Varieties*	Simplifies the application procedure and shortens the commercialization time for GM crops.
	Amends *Measures of Approval of Production and Operation for Crop Seeds*	
	Amends *Regulations on Naming Agricultural Plants*	
	MOA: Issues *Guide for Biosafety Measurement of Gene Edited Crops*	Issues regulations on gene edited crops for the first time in history. Simplifies the administration and shortens the approval time for commercialization of gene edited crops.

Source: Authors compilation according to various sources of documents and materials.

### 3.1 Stage I: Building up a Regulatory Framework Prior to Launching Biotech R&D (1978 – 85)

In the early period, the government began to put in place a framework for state-led biotechnology research and development. At the same time, it also began to establish a regulatory system of formal biosafety regulations to be applied to GMO-crops developed domestically.

In 1978, the Ministry of Science and Technology (MOST) issued the *National Science and Technology Development Program Outline (1978–85)* to garner the national resources required to support eight major science fields including agriculture. The strategy for developing agricultural biotechnology included three major facets. First, to develop new seed varieties with high productivity, high quality, and pest resistance. Second, develop novel breeding theories and technical methodologies. Third, to encourage theoretical and applied work based on genetic improvements using agricultural germplasm.

In 1985, MOST, State Development and Planning Commission (SDPC, *Jiwei*), and State Economic and Trade Commission (SETC, *Jingmaowei*) jointly composed a draft of a *National Biotechnology Development Policy Outline*, which was issued by General Office of the State Council (GO)^2^^2^GO is the executive institute which is responsible for day-to-day operations of the State Council. in 1988. The *Outline* emphasized developing high-quality, high-yield, and disease and pest resistant varieties through the use of biotechnological methods. The *Outline* also pointed out that biotechnology legislation needed to be put onto the political agenda.

### 3.2 Stage II: Initiate Biotechnology Research & Development (1986 – 1992)

The processes that lead to the commercialization of GM crops requires a long-term commitment, which needs ongoing investments in research and development (R&D) and a well-developed institutional system of biosafety administration. Since the mid-1980s, the Chinese government has issued policy outlines as national guidance for development efforts and has committed to an ambitious budget for biotechnology R&D. Unlike developed economies, China’s agricultural biotechnology R&D has been accomplished through public-sector-dominated investment targeted at building up a state-owned research pipeline for GMOs.

In March 1986, Deng Xiaoping approved a new research program – the National High-tech Research and Development Plan (863 Plan). The objective of the 863 Plan is to improve China’s science and technology research ability in the seven high-tech fields (including biotechnology, space technology, information technology, and others) so as to compete with the most advanced technologies in the world. Consequently, six new National Key Laboratories dealing with biotechnology and molecular biology research were established in north, central, and south China.^35^ The program was originally designed to run for 15 years and provide RMB 10 billion (US$ 30.1 billion)^3^^3^In 1986 the currency conversion was 1 RMB = 3.2 US$. for all seven fields. Roughly RMB 1.5 billion (US$4.8 billion) was allocated to biotechnology and RMB 0.8 billion (US$ 2.5 billion) for GM crop research.^10^

### 33 Stage III: Develop Bio-Administration System and Prepare to Promote Commercializing GM Crops (1993 – 2009)

On December 24, 1993, the first formal regulation for the administration of genetic engineering pertaining to biosafety (Measures for the Safety Administration of Genetic Engineering, SAGE) was promulgated by the State Science and Technology Commission (SSTC) under MOST. From that point on, the government began to formulate biosafety regulations, along with making hefty investment in R&D, to establish the formal regulatory setting for the commercialization of GMOs. The extent of the risks to human beings was taken into account. Genetic engineering activity was classified into four classes: animals, plants, microorganisms and the ecological environment. It also regulated genetic engineering’s safety evaluation, application and approval, safety control measures, and legal responsibilities [State.^36^]

On July 10, 1996, following MOST, the MOA promulgated *Decree 7–Implementation Measures for the Safety Administration of Agricultural Genetic Engineering* (*SAAGE*) – on biosafety administration for agricultural crops and animals. It enabled the MOA to conduct biosafety evaluations by establishing the Administration Office for Biosafety of Agricultural GMOs (AO) and Administration Committee for Biosafety of Agricultural GMOs (AC).

In March 1997, the government launched another important research program – the National Plan on Key Basic Research and Development (973 Plan). It was designed to support basic research by funding a range of programs in the field of agriculture, energy, information, human health and resources and environment. Life sciences and biotechnology were key areas of support garnering US$238 million^37^ out of the total budget of US$302.^10^

In 1997 and 1998, the government authorized the commercial production of four GM crops ‒ Bt cotton, tomatoes, sweet peppers, and petunias. There was no discernable opposition.

In 1999, the MOST initiated the *Special Foundation of Transgenic Plants Research and Commercialization (SFTPRC)* with a total budget of US$60 million over 1999–2003.^10^ The objective is to promote both research and commercialization of GM crops to fully implement the goals set out in the 863 and 973 plans.

On December 1, 2000, for the first time, the administration of biosafety for GM plants was included in a national law, the *China Seed Law*, passed by National People’s Congress. It emphasized that the breeding, experiment, examination, and extension work for GM plants must be conducted within the safety-evaluation framework, and that strict safety control measures are required.^38^

On May 23, 2001, the State Council promulgated *Regulations on Administration of Agri- cultural GMOs Safety (RAAGS)* (*Decree* 304). This Decree applies to animals, plants, microorganisms and their products. More important, this decree allows the State Council to establish an Inter-Ministerial Joint Conference for Administration of Agricultural GMOs Safety (IMJC, *bujilianxihuiyi*), and the MOA sets up two organizations: Administration Office for Biosafety of Agricultural GMOs (AO, *anquanguanlibangongshi*) and Administration Committee for Biosafety of Agricultural GMOs (AC, *anweihui*). RAAGS sets out that the biosafety examination of agricultural GMOs is a stage-by-stage evaluation system. The applying unit can only move to the next stage contingent on the approval of the previous stage by AC. Agricultural GMOs are classified into Class I, II, III, and IV regarding the extent of their risks to human beings, animals, plants, microorganisms and the ecological environment. Agricultural GMOs listed in the labeling catalog must be labeled when they are sold within China.^39^

On January 18, 2002, the MOA promulgated *Decrees 8, 9*, and *10*. These three decrees constitute the body of the current biosafety administration system for agricultural GMOs, although they were slightly amended in 2004 and 2016 respectively. These three decrees regulate agricultural GMOs from three perspectives: biosafety evaluation, biosafety administration for imports, and labeling. It is worth noting, GM products selected for labeling were required to show a clear label, otherwise they would be banned from being imported into, and sold, in China.^40^ The MOA is responsible for labeling examination and enforcement, monitoring and administration. The first batch of GMOs with mandatory labeling required, all imported, were released in 2002, including five categories and 17 products, such as GM soybean, maize, canola, cotton seeds, and tomatoes [Administration Office for Biosafety of Agricultural GMOs.^41^]

Over the period 2004–2006, after the major administration body was up and running, two national laws to regulate labeling requirements for GMOs were put in place. In August 2004, NPC amended the *China Seed Law*, which mandates labeling for GM seeds. In November 2006, NPC promulgated the *Law of Quality and Safety for Agricultural Products of China (LQSAP)* which requires that agricultural GMOs should be labeled in accordance with relevant regulations on biosafety administration. Furthermore, the mandatory labeling requirement was also emphasized in the Number-One Central Document of 2007.^4^^4^China’s central government normally indicates the country’s most important issues and the direction of development strategies in the No. 1 Document of the Central Committee of Communist Party of China (CPC) and the State Council.

On February 9,,^14^ the State Council promulgated *The Medium and Long-term Plan for the Development of Science and Technology: 2006–2020 (MLPST)*. It identifies several areas as development priorities, including 16 major national projects (*zhongda zhuanxiang*), 27 frontier technologies in 8 areas, 18 basic-science research questions, and 4 major scientific research plans. Biotechnology is specified as one of the frontier technologies. In the area of agricultural science and technology, MLPST clearly expressed the government’s desire to become the world leader in agricultural technology with an objective to improve agricultural productivity. The end goal was to achieve food self-sufficiency within 15 years.^14^

On April 8, 2007, the National Development & Reform Commission (NDRC) promulgated *The 11th Five-year Development Plan for Biology Industry (2006–2010)*. The plan included an expectation that China would garner RMB 2000 billion (US$6.2 billion)^5^^5^In 2007 the currency conversion was 1 RMB = 3.1 US$. in added value from the biology industry. The industry was projected to be four percent of the GDP by 2020. In the area of agricultural biology, 100–150 new agricultural varieties were forecast to be approved for production over the period of the 11th Five-Year Plan period.

In 2008, as one of 16 major national projects, the Major Breeding Project of GMOs Varieties (GMMP) was launched and the aggregate public R&D budget for GM biotechnology was expected to reach US$3.8 billion over 2008–20.^14^

On June 1, 2009, the National People’s Congress promulgated the China Food Safety Law, in which, for the first time, GM food safety was included in a national law. By this time, the government believed that it was time to commercialize GM rice and maize given that the technology was considered mature and well-prepared regulations and a functioning administration system was in place.

On August 17, 2009, the MOA issued production safety certificates to two GM major crops: Bt rice (Huahui-1 and Xianyou-63) and phytase maize (BVLA430101). Production safety certification means that R&D activities for a GM crop have been completed, and the applicant is cleared to apply for release of the variety for commercial production. Once an applicant receives a production safety certificate, it still needs to apply for commercialization from the government. The issuing of the product safety certificates for these major GM rice and maize varieties suggests that the central government was determined to push the commercialization of GM major crops to the next level.

By the end to the first decade of the 21^st^ century the regulatory and administrative stage was set for China to become a major producer of GM crops. As seen above, the government had been measured and meticulous in constructing the means to manage production agriculture and consumer safety when GM crops began to be commercially grown and entered food distribution channels.

Like the US, the design of the Chinese regulatory system is pro-technology and science-based. It makes no formal provisions for nonscientific objections to the commercialization of GM crops or GM products entering the food supply chain. It does differ from the US approach in that it requires GM foods to be labeled, thus allowing consumers the option of not buying GM crops if that is their preference.^42,43^ There is no labeling requirement in US regulations although attempts have been made to require labeling in individual US states and municipalities.

The design of the Chinese regulatory system is very different from that of the EU which is precautionary and skeptical of science.^16^ The EU regulatory system also makes provision for nonscientific objections to biotechnology to be incorporated into its decision-making processes.^18^ Science only informs decisions but can be over-ruled by Member States. Labeling is required in the EU but is largely redundant because production and imports for human consumption are, in effect, banned.

### 3.4 Stage IV: Delays in Commercializing GM Major Crops (2010 and Afterward)

Despite their apparent enthusiasm for expanding the use of GM seed, the government issued no production safety certificates for GMOs from 2009 to 2018. Furthermore, it has made no progress in commercializing Bt rice (Huahui-1 and Xianyou63) and phytase maize (BVLA430101) that received the production safety certificates in 2009. After waiting for 10 years for commercialization in China’s domestic market, Huahui-1 has been approved by U.S. Food and Drug Administration (FDA) for marketing in 2018.^44^ After a decade of inaction on issuing biosafety certificates, the MOA issued production safety certificates to IR and HT maize (DBN9936 and Ruifeng125) and HT soybean (SHZD3201) in December 2019. On July 15, 2020, the MOA issued production safety certificates to HT maize DBN9858 and HT soybean (ZH6106 and DBN9004). In January 2021, the MOA issued production safety certificates to IR and HT maize (DBN3601T, i.e., DBN9936× DBN9501) and IR maize (ND207 and Ruifeng8). At the time of writing, however, there has been no announcement regarding the initiation of commercialization.

The decade of inaction on commercialization was one of intense debate carried out in the press – in the open and not obviously limited by the government – and on social media. Staring with rumors and scandals, the discussion evolved into a number of influential debate themes pushed by anti-GMO activists such as Greenpeace. These were specifically designed to appeal to morality and patriotism present in Chinese society. The theme’s development started with connecting GMOs to *food safety* concerns. It then progressed to discussions of the *right-to-know* about GMOs in the food system and the *right-to-choose* not to consume GMOs. Moreover, the anti-GM camps propounded a *conspiracy theory* that appealed to citizen’s patriotism. The claim that there was a *relative inequality* in the accessibility to *safe food* (non-GMO) between officials and the general public added to some of public anger over the commercialization decision. As a result, the commercialization of GMOs evolved from a scientific issue to a moral and political debate.

As in the EU (and in the US), the anti-GMO activists are strident and have strongly held preferences. What is unknown is how much support there is among civil society in China. What is opaque is the channel by which the concerns expressed regarding agricultural biotechnology by anti-GMO activists (and broader civil society) can influence government decision-making. The inaction on commercialization can be clearly seen but the government is silent about its inaction. Investment into research and development on agricultural biotechnology continues uninterrupted. The scientific establishment remains largely supportive, but decisions are now in the political realm. Thus, in China, GMOs are now being dealt with at the political level as they are in the EU.^18^

China’s government cares deeply about social cohesion.^45^ Any issues that can potentially lead to social discord among the populace are seen as a threat. Regarding issues that might threaten social cohesion, the Chinese government takes a precautionary approach. Unlike the US where private sector agribusiness firms that have invested in agricultural biotechnology act as a counterbalance to the anti-GMO activists through their legally sanctioned ability to lobby politicians and government officials, formal lobbying is not common in China. For the seed industry, state-owned enterprises (SOE) are more active in lobbying than private firms.^46^ Private companies appear to play a very limited role in China’s policy-making process.^47^ As major investment in research and development in agricultural biotechnology is made by the state, the research establishment is a bystander in the process.^6^^6^In the EU, having lost the initial debate over regulation of GMOs – the research establishment was largely unprepared for the backlash^17^^–^ most large agribusiness firms have moved their research and development divisions to the US^8^ so that advocates of GMOs only try to influence policy making from outside. Their vested interest is in maintaining the research establishment, where they appear to have been successful, but not commercialization. The combination of social coherence-based precaution and an absence of pro-GMO commercialization advocates has led to a political decision whereby GMO technology is eschewed despite deep-rooted concerns over food security.

After more than a decade of silence about the commercialization, the MOA has promulgated a number of policies and regulations since the beginning of 2022 with the objective of opening a path for commercialization of major GM crops. On January 21, 2022, the MOA amended four major regulations regarding GM crop administration (Table 2). These adjustments of regulations simplify the application procedure of GM crops and shorten the time for commercialization. On January 24, 2022, the MOA issued *Guide for Biosafety Measurement of Gene Edited Crops* to regulate gene edited crops for the first time in history.^7^^7^The MOA defines gene edited crops as GM crops with no external genes. It simplifies the application process and shorten the approval time for gene edited crops for commercial production compared to GM crops. China has successfully developed gene-edited rice, maize, wheat, and soybeans. The *Guide* is expected to speed up the commercialization of gene edited crops.

## 4. Conclusions

The global agricultural system has become increasingly fragile with the challenges of climate change, resource limitation, population growth, economic slowdown and regional conflicts, as well as public health crises (such as the COVID-19 pandemic). How to transform the agricultural system to be more resilient and sustainable has become a major policy issue in China and across the world.

China’s agricultural output has grown dramatically with the average growth rate of 4.6% over the last three decades.^48^ However, since 2003 China has turned into a net food importer and the import-export gap has been increasing ever since.^4^ Soybean accounts for more than 70% of total imported food crops.^4^ China is the world largest soybean importer, accounting for over 60% of the world imported soybean.^4^ The net import quantity has kept increasing dramatically since late 1990s and reached record high of more than 100 million tons in 2020.^4^ Soybean yield in China, however, is lower than the average worldwide yield and the gap has kept increasing since 2003.^4^

China is the second-largest maize producer in the world, accounting for more than 20% of the global maize production.^4^ The maize yield in China, however, is only about 56% of the US – the world’s highest yield – over the last two decades.^4^ The rapid growth of maize production has been outstripped by growth in maize demand due to the increased demand for meat as per capita income increased. China became a net maize importer in 2009 and net imports reached the historically high level of more than 11 million tons in 2020.^4^ The increasing trend of maize imports is predicted to continue over the next decade.^49^

One strategic objective of China’s government is to harness biotechnology to help achieve national food security goals. China has made a consistent and significant R&D investment in biotechnology over the last decades.^9,10^ Despite these investments in domestic development of GM crops – GM soybean, maize and rice China has commercialized no major crops.

It is clear that China’s story on commercialization of GMOs is not yet complete. Which way will government policy develop? With the rapid growth of imports of maize and soybean, in recent years the government has begun to prepare for the commercialization. It has finally resumed the issuing of production safety certificates, to domestically developed maize and soybean varieties over the last 3 years. Meanwhile, the government amended the regulations regarding the administration and commercialization of GM crops and gene edited crops in 2022 to pave the way for commercialization of major GM crops. Rice, with the strong opposition from the public and a self-sufficient supply in 2019, is not, however, expected to be commercialized in near future. While in the case of maize, with less resistance from the public given that it is primarily used as livestock feed and the recent increase in imports^50^, one might expect the government to commercialize GM maize in near future. In spite of the relatively high price of non-GM soybean, domestic production represents only a small proportion of total quantity demanded (import quantity is six to seven times domestic production, Huang, 2021), the commercialization of soybean seems further off in the future.
